# Perceived Satisfaction, Quality of Care, and Enablement Among Patients Receiving Acute Care From Nurse Practitioners in Taiwan: A Multi-Hospital Study

**DOI:** 10.1097/jnr.0000000000000764

**Published:** 2026-08-05

**Authors:** Ying-Ru CHEN, Shiow-Luan TSAY, Ling-Chun LU, Pei-Chun TSAI, Hui-Ling YEH, Chin-Huang CHEN, Jia-Ying HU, Fong-Ping TANG, I-Ping HUANG

**Affiliations:** 1 School of Nursing, National Taiwan University, Taipei, Taiwan; 2 Department of Internal Medicine, National Taiwan University Hospital, Taipei, Taiwan; 3 College of Nursing and Health Sciences, Da-Yeh University, Changhua, Taiwan; 4 Second Degree Program of Bachelor of Science in Nursing, College of Medicine, National Taiwan University, Taipei, Taiwan; 5 Department of Nursing, E-Da Hospital, Kaohsiung, Taiwan; 6 Department of Nursing, Hualien Tzu Chi Hospital, Buddhist Tzu Chi Medical Foundation, Hualien, Taiwan; 7 Department of Nursing, Ditmanson Medical Foundation Chia-Yi Christian Hospital, Chiayi, Taiwan; 8 Department of Nursing, Yuan’s General Hospital, Kaohsiung, Taiwan; 9 Department of Nursing, Taipei Municipal Gan-Dau Hospital, Taipei, Taiwan

**Keywords:** nurse practitioners, patient satisfaction, quality of health care, patient enablement, acute care settings

## Abstract

**Background:**

Nurse practitioners (NPs) have increasingly been employed in acute care hospitals to help reduce shortages in medical staff. However, no data exist regarding the outcomes of hospitalized patients in Taiwan receiving continuous NP care.

**Purpose:**

This study was designed to investigate perceived satisfaction, quality of care, and enablement among patients receiving care from NPs in Taiwan.

**Methods:**

A cross-sectional design was used to investigate a sample of adult patients admitted to medical or surgical units in participating hospitals in Taiwan between August 1, 2023, and June 30, 2024. Patients who met the inclusion criteria were invited to complete the study questionnaire during hospitalization. Data on basic institutional and patient characteristics were collected. The modified Patient Enablement and Satisfaction Survey was used to investigate perceived satisfaction, quality of care, and enablement among the participants, and multivariable linear regression was applied to explore the potential factors associated with these three variables.

**Results:**

Seven hundred eight medical and surgical patients from three medical centers and seven regional hospitals across Taiwan participated in this study. Perceived satisfaction with NP care in the sample ranged from satisfied to very satisfied. The regression analysis revealed that ratio of NPs to acute care beds, area of residence, admission route, medical division, and perceived health status were significant predictors of patient satisfaction with NP care (*p*<.05); ratio of NPs to acute care beds, acute illness severity, medical division, and self-perceived health status were significant predictors of quality of care (*p*<.05); and ratio of NPs to acute care beds, acute illness severity, admission route, medical division, and perceived health status were significant predictors of patient enablement (*p*<.05).

**Conclusions/Implications for Practice:**

The findings of this study indicate that the general level of satisfaction among medical and surgical patients in Taiwan with NP care is relatively high. Numerous factors were identified as key influencers of perceived satisfaction, quality of care, and enablement among medical and surgical patients. The results of this study provide vital information to help NPs and hospital administrators develop and implement strategies to improve satisfaction, quality of care, and enablement among patients receiving acute care from NPs.

## Introduction

Taiwan’s national legislature implemented an education, licensure, and practice model for nurse practitioners (NPs) in 2006 with the goal of significantly addressing the shortage in health care providers and the rising demand for acute care services among the general population. Since then, the number of NPs has grown rapidly to over 11,000, all of whom are partnered with physicians in tertiary hospitals (Taiwan Association of Nurse Practitioners, 2025). Taiwan regulations require that NPs must be supervised by designated physicians and provide care to patients in accordance with authorized protocols. Despite these restrictions, NPs strive to maintain their professional autonomy in patient care. NPs in acute care institutions provide hospitalized patients with direct health care, collaborate with care teams, educate patients and their families, monitor care quality, and demonstrate leadership and professionalism (Ho et al., 2021; Hu et al., 2025; Taiwan Association of Nurse Practitioners, 2025; Tsay et al., 2023).

Hospitals must monitor the contributions of NPs within the health care system as more NPs enter the workforce, with these data providing empirical evidence that may be used to guide policy development. Outcome measures related to NP performance frequently include length of hospital stay, adverse events, infection rates, hospital readmissions, procedural complication rates, hospital mortality, and patient/family satisfaction (Barnett et al., 2022; Kapu, 2021; O’Reilly et al., 2021). However, these outcomes reflect the collaborative effort of physicians, NPs, and other providers, thus providing only limited information on the unique contributions of NPs.

A systematic review of the literature on NP care outcomes from 2008 to 2018 confirmed the value of continuity of care, quality and safety metric improvement, patient and staff satisfaction, and the educational experiences of patients, residents, and fellows (Kahn et al., 2015; Kleinpell et al., 2019). However, these outcomes have frequently been measured using a single item such as “How satisfied are you with the care provided by NPs?” Consequently, the findings of these studies cannot sufficiently inform efforts to improve patient care. Key outcomes must be measured using psychometrically validated instruments for results to serve as evidence in support of the contributions of NPs to health care services.

To overcome these limitations in the measurement of NP care outcomes, Desborough et al. (2014, 2016) developed the Patient Enablement and Satisfaction Survey (PESS), with items scored on a 20-item instrument with Likert-type item. However, the psychometric properties of the PESS were not established during instrument development. O’Reilly et al. (2021) investigated the construct validity of the PESS across the three dimensions of satisfaction, quality of care, and patient enablement and, to date, only two small sample studies have used the PESS to investigate patient satisfaction, quality of care, and enablement (Desborough et al., 2016; O’Reilly et al., 2021).

Patient satisfaction with care, among the most frequently reported clinical outcomes, is regularly employed as an indicator of treatment compliance (World Health Organization, 2025). Patients have reported high satisfaction with NP-administered care (Aiken et al., 2021; Kippenbrock et al., 2019; O’Reilly et al., 2021). A systematic review of 13 randomized controlled trials on the relative effectiveness of NPs compared with physicians revealed NPs to have a relatively more positive effect on self-perceived health status and care satisfaction among pediatric and adult patients than physicians (Htay & Whitehead, 2021). In addition, a secondary data analysis of the U.S. Consumer Assessment of Healthcare Providers and Systems by Kippenbrock et al. (2019) found higher satisfaction ratings for NPs than physicians among patients.

Quality of care represents the degree to which health services increase an individual’s likelihood of achieving desired health outcomes (World Health Organization, 2025). The U.S. Institute of Medicine lists six goals for quality of care: safety, effectiveness, patient-centeredness, timeliness, efficiency, and equity (Institute of Medicine, 2001). Safety refers to the protection of patients from harm throughout their treatment journey. Effectiveness refers to whether health care services produce the desired outcomes. Patient-centeredness refers to the integral role of the patient and the patient’s family in decision-making regarding care and treatment. Timeliness refers to the delivery of health services at the appropriate moment. Efficiency refers to delivering the best care at the lowest cost. Finally, equity refers to equality in health care access for all individuals. Desborough et al. (2014) defined quality of care as self-perceived by the patient, and influenced primarily by their care plan and related treatment decision-making.

Patient enablement encompasses the ability of a patient to understand, manage, and cope with illness in accordance with the care provided by NPs, who promote the ability of patients to control their health (Desborough et al., 2014; O’Reilly et al., 2021). The concepts of enablement and empowerment have often been used interchangeably. NPs routinely practice patient-centered care in hospital settings, empowering patients to manage their illness through support, guidance, education, and consultations (van Dusseldorp et al., 2019). The adaptation of the PESS in Australia reveals that patients receiving care from NPs perceive high levels of enablement (Desborough et al., 2016; O’Reilly et al., 2021).

Patient satisfaction, quality of care, and enablement are key outcome measures of NP care delivery and patient-perceived empowerment resulting from increased understanding of and capacity to self-manage their illness. These outcomes have been identified as robust indicators of the quality of services provided by NPs (Desborough et al., 2014). Patients are more likely to comply with treatment regimens when services are perceived as satisfactory.

Numerous factors influence patient satisfaction, quality of care, and enablement when receiving care from NPs. First, staffing patterns may affect outcomes of NP care. Hospitals with higher NP/bed ratios have reported greater care quality and safety (Aiken et al., 2021). Registered nurses who work closely with NPs reported lower burnout, higher job satisfaction, and higher intention of staying in their jobs (Aiken et al., 2021). Thus, hospitals with more NPs can benefit patients, staff nurse satisfaction, and care quality, indicating that NPs add value for hospitals, patients, and other nurses. Second, institution, patient, and clinical characteristics may influence care outcomes. Thus, demographic variables, health care institution type, area of residence, admission route, medical division, and patient clinical severity, length of hospital stay, and perceived health status were incorporated in this study as covariates based on theoretical rationale and prior empirical evidence (Kleinpell et al., 2019; O’Reilly et al., 2021). However, the minimal scholarly investigation into the effect of these factors on NP practice outcomes warrants further research.

The review of the literature on NP care outcomes conducted in this study revealed that most existing studies included small sample sizes and targeted patients from local community clinics, with no studies conducted in Taiwan. In addition, few scholars have examined NP-related factors influencing patient outcomes in acute care settings. Thus, this study was designed as a multi-hospital survey using a validated instrument to investigate perceived satisfaction, quality of care, and enablement among patients receiving care from NPs.

### Conceptual Framework

The conceptual framework developed by Sidani and Doran (2010) was adapted in this study to evaluate the outcomes of NP involvement in acute care. This framework encompasses the complex factors present in acute care settings across the three major components of structure, process, and outcomes. The structural component includes variables related to the patient (i.e., demographic characteristics, acute illness severity, and comorbidities) and organization (i.e., number of NPs on staff and type of health care institution). The process component pertains to the health service practices of acute care NPs. NPs not only provide direct care but also explain health conditions and treatment plans to their patients. Importantly, NPs provide patients with information on their condition and guidance on ways to self-manage their illness. The outcome component includes the degree of patient satisfaction, quality of care, and patient enablement during self-management.

## Methods

### Study Design

A cross-sectional study design was used in this study, and 95 invitations to participate were mailed to hospital NP managers. Three medical centers and seven regional hospitals responded and agreed to participate. Patients who were receiving care from NPs, met the inclusion criteria, and agreed to participate were asked to complete the consent form and questionnaire during their hospitalization. Basic data on each health care institution and patient were collected, and the PESS was administered to investigate their perceived satisfaction, quality of care, and enablement. The study protocol was approved by an institutional review board.

### Participants

Patients aged 18 years or older, who were admitted to either a medical or surgical ward of the participating health care institutions, and who had received care from NPs for at least 3 days, were eligible for inclusion. Patients who were not in stable condition, unable to communicate, had a psychological disorder, were involved in medical disputes, or planned to be automatically discharged were excluded. A power analysis conducted in G*Power 3.1.9.7 (Faul et al., 2009) determined a minimum sample size of 152 was needed given a medium effect size value of 0.15, statistical power of 0.90, significance level of .05, and 10 potential predictors.

To facilitate reliability, statistical confidence, and generalizability, data from 708 participants were collected and included in the analysis. The study protocol was reviewed and approved by the IRB (No. 202310091RINC).

### Data Collection

Data were collected between August 1, 2023, and June 30, 2024, after ethical approval and patient consent were obtained. The questionnaire was administered through the iPad online SurveyCake system. Eligible patients were asked to fill out the questionnaire on a research team–provided iPad during hospitalization at least 1 day before discharge.

### Measures

Data were collected in accordance with the structures, processes, and outcomes incorporated into the conceptual framework described in the study by Sidani and Doran (2010). Patient and organization variables were collected at the structural level, with the former including age, gender, educational level, diagnosis, acute illness severity (based on National Early Warning Score [NEWS] 2 criteria), Charlson Comorbidity Index (CCI) score, and perceived health status. NEWS 2 and CCI scores were calculated and collected by one of the NP researchers in this study. The organizational variables included the type of health care institution, the number of acute beds, and the number of NPs employed. To measure process-related variables, the number of hours NPs were engaged in direct patient care, and the number of patients for whom they were responsible during an 8-hr shift were recorded. The outcome variables were quality of care, patient satisfaction, and patient enablement during self-management.

NEWS 2, a metric commonly used to identify acute illness severity and clinical deterioration, is based on scores for physiological parameters such as respiratory rate, supplemental O₂, body temperature, blood pressure, and consciousness. Each condition is assigned a score, all of which are summed to obtain a total score, with total scores below 5 indicating the respondent is relatively stable and low risk; scores between 5 and 7 indicating medium stability, greater risk, and recommended referral for treatment; and scores ≥ 7 indicating high risk and the need for immediate intervention (Smith et al., 2019).

Patient experience with the care provided by acute care NPs was evaluated in this study using the modified PESS (PESS-ANP; O’Reilly et al., 2021). The developers of the original PESS (Desborough et al., 2014) divided the instrument into two main sections, that is, patient satisfaction and patient enablement. As the original PESS was tested for face validity only, O’Reilly et al. (2021) investigated its psychometric property and modified the original 20-item PESS by deleting one item (Item 14) due to low factor loading, thus revising the PESS into a 19-item scale with items scored on a 5-point Likert scale. The construct validity of this modified PESS was evaluated using exploratory factor analysis. The three-factor structure of patient satisfaction, patient enablement, and quality of care explained 69.4% of the overall variance. The internal consistency (i.e., reliability) of the PESS scale was assessed based on Cronbach’s α, with the .95 value surpassing the required minimum of .90.

### Data Analysis

SPSS Statistics 24.0 (IBM Corp., Armonk, NY, USA) was used for the analysis, with continuous variables summarized using mean and *SD*, and categorical variables summarized using frequency and percentage. Also, multivariable linear regression analysis was used to examine the effects of structural and process variables on the outcomes.

Multivariable linear regression models were constructed to identify predictors of patient satisfaction, quality of care, and patient enablement. For multivariable regression analyses, potential confounders were identified a priori. Variables identified as *p*<.1 in the univariate analyses were considered candidates for entry and, in accordance with a purposeful selection strategy, variables of established clinical relevance were retained regardless of statistical significance (Chowdhury & Turin, 2020; Vittinghoff et al., 2005). This approach ensured appropriate adjustment for confounding and strengthened the validity of the findings. To reduce residual confounding, all of the models were adjusted for institutional and clinical characteristics, including health care institution type, area of residence, admission route, medical division, and patient clinical severity (as measured using the NEWS 2 score). In addition, demographic variables (age, sex, and educational attainment), length of hospital stay, and perceived health status were incorporated as covariates based on theoretical rationale and prior empirical evidence. Thus, across all of the models, both institutional and patient-level factors were comprehensively accounted for to ensure the robustness and clinical relevance of the findings.

### Ethical Approval

This study was approved by the Ethics Committee of National Taiwan University Hospital (202310091RINC). All participants were provided with both oral and written explanations of the study and provided informed consent to participate.

## Results

### Demographic and Clinical Characteristics

This multi-hospital study included 708 patients hospitalized in 10 major institutions (three medical centers and seven regional hospitals) who received care from 495 NPs (Table 1). The majority (*n* = 447; 63.14%) were recruited from regional hospitals. Participant mean age was 58.60 (*SD* = 15.42) years, and 60.73% (*n* = 430) were male. Nearly two-thirds (*n* = 428, 60.45%) had completed high school or higher education, and most were from either southern (*n* = 336, 47.46%) or northern (*n* = 201, 28.39%) Taiwan. The demographic characteristic information collected in this study is summarized in Table 1.

**Table 1:** Participant and Hospital Characteristics (N = 708)

Variable	*n* (%)
Acute care beds (*M* and *SD*)	583.59 (282.17)
NPs per hospital (*M* and *SD*)	107.50 (51.76)
NP-to-acute-care-beds ratio (*M* and *SD*)	0.18 (0.04)
Institution	
Medical center	261 (36.86)
Regional hospital	447 (63.14)
Patient characteristics	
Age (years; *M* and *SD*)	58.60 (15.42)
Sex	
Male	430 (60.73)
Female	278 (39.27)
Educational level	
Elementary school or below	150 (21.19)
Junior high school	130 (18.36)
High school	209 (29.52)
College or above	219 (30.93)
Area of residence within Taiwan	
North	201 (28.39)
Central	84 (11.86)
South	336 (47.46)
East	87 (12.29)
NEWS 2 (*M* and *SD*)	0.97 (1.46)
CCI (*M* and *SD*)	5.76 (3.59)
Body mass index (*M* and *SD*)	24.64 (9.27)
Admission route	
Outpatient clinic	422 (59.60)
Emergency room	286 (40.40)
Medical division	
Surgical department	454 (64.12)
Internal medicine	254 (35.88)
Length of hospital stay (*M* and *SD*)	9.24 (9.53)
Perceived health status (*M* and *SD*)	3.26 (1.03)

*Note.* NPs = nurse practitioners; NEWS 2 = National Early Warning Score 2. CCI = Charlson comorbidity index.

The NP-to-acute-care-beds ratio was 0.18 (*SD* = 0.04), and the participants had a mean NEWS 2 score of 0.97 (*SD* = 1.46) and a mean CCI of 5.76 (*SD* = 3.59). Over half of the participants (*n* = 422; 59.60%) had been admitted through outpatient services, while 40.40% (*n* = 286) had been admitted through the emergency department. Two-thirds (*n* = 454; 64.12%) had been admitted to the surgical department, and 35.88% (*n* = 254) had been admitted to the internal medicine service. The mean hospital stay length was 9.24 (*SD* = 9.53) days, and the average self-perceived health score was 3.26 (*SD* = 1.03, range = 1–5; Table 2).

**Table 2:** Relationships Among Demographic Characteristics, Satisfaction, Quality of Care, and Enablement (N = 708)

Variable	Patient Satisfaction			Quality of Care			Patient Enablement		
	*M* (*SD*)	*t/F/r*	*p*	*M* (*SD*)	*t/F/r*	*p*	*M* (*SD*)	*t/F/r*	*p*
Total acute care beds ^a^	583.59 (282.17)	.08	.837	583.59 (282.17)	.05	.153	583.59 (282.17)	.04	.351
Total NPs ^a^	107.50 (51.76)	.02	.577	107.50 (51.76)	.03	.433	107.50 (51.76)	.02	.654
NP-to-acute-care-beds ratio ^a^	0.18 (0.04)	.12	.002	0.18 (0.04)	.08	.026	0.18 (0.04)	.07	.074
Institution ^b^		3.42	<.001		4.95	<.001		4.24	<.001
Medical center	44.40 (5.80)			17.56 (2.59)			22.09 (3.29)		
Regional hospital	42.73 (6.50)			16.48 (3.15)			20.91 (3.72)		
Age ^a^	58.60 (15.42)	−.06	.114	58.60 (15.42)	−.08	.031	58.60 (15.42)	−.10	.008
Sex ^b^		−1.22	.225		−1.40	.163		−1.95	.051
Male	43.11 (6.30)			16.75 (3.03)			21.14 (3.70)		
Female	43.70 (6.29)			17.08 (2.93)			21.68 (3.45)		
Educational level ^c^		1.10	.348		1.35	.257		2.58 ^d^	.053
Elementary school or below	43.05 (6.58)			16.62 (3.13)			20.96 (3.79)		
Junior high school	42.62 (6.77)			16.58 (3.30)			20.88 (4.00)		
High school	43.56 (6.18)			17.01 (2.85)			21.46 (3.30)		
College or above	43.77 (5.90)			16.88 (3.00)			21.81 (3.50)		
Area of residence ^c^		9.38 ^d^	<.001		11.98 ^d^	<.001		7.34	<.001
① North	44.22 (5.79)	①>②		17.26 (2.68)	④>①		21.81 (3.41)	①>③	
② Central	41.65 (7.99)	①>③		16.41 (3.74)	①>③		20.86 (4.13)	②<④	
③ South	42.62 (6.14)	②<④		16.39 (3.01)	②<④		20.88 (3.63)	③<④	
④ East	45.75 (5.21)	③<④		18.32 (2.20)			22.63 (3.04)		
NEWS 2 ^a^	0.97 (1.46)	−.09	.015	0.97 (1.46)	−.16	<.001	0.97 (1.46)	−.15	<.001
CCI ^a^	5.76 (3.59)	−.03	.402	5.76 (3.59)	−.03	.452	5.76 (3.59)	−.04	.359
BMI ^a^	24.64 (9.27)	.01	.793	24.64 (9.27)	−.01	.897	24.64 (9.27)	.02	.685
Admission route ^b^		4.14	<.001		3.15	.002		4.43	<.001
Outpatient clinic	44.17 (5.81)			17.18 (2.75)			21.86 (3.24)		
Emergency room	42.14 (6.79)			16.44 (3.29)			20.60 (4.00)		
Medical division ^b^		−2.90	.004		−2.53	.012		−2.70	.007
Surgical department	42.85 (6.40)			16.67 (3.09)			21.09 (3.71)		
Internal medicine	44.24 (6.02)			17.25 (2.79)			21.83 (3.39)		
Length of hospital stay ^a^	9.24 (9.53)	.07	.069	9.24 (9.53)	.01	.748	9.24 (9.53)	−.02	.682
Perceived health status ^a^	3.26 (1.03)	.13	<.001	3.26 (1.03)	.13	<.001	3.26 (1.03)	.14	<.001

*Note.* NPs = nurse practitioners; NEWS 2 = National Early Warning Score 2; CCI = Charlson comorbidity index; BMI = body mass index.

^a^ Pearson’s correlation coefficient; ^b^ Independent *t* test; ^c^ One-way analysis of variance (Scheffe’s post hoc test); ^d^ Variance unequal (Robust analysis of variance with Games-Howell method).

### Relationships Among Demographic Characteristics, Satisfaction, Quality of Care, and Enablement

The participants self-identified as being satisfied to very satisfied with the care received from NPs, with a mean item-level satisfaction score of 4.33 (*SD* = 0.63, range = 1–5). Those in medical centers reported significantly higher satisfaction, quality of care, and enablement compared with their peers in regional hospitals (*p*<.001). The NP-to-acute-care-beds ratio was found to correlate positively with both patient satisfaction (*p*<.01) and quality of care (*p*<.05), supporting that greater numbers of NPs on staff improve patient outcomes. An inverse association was found between age and both quality of care (*p*<.05) and enablement (*p*<.01). Regarding regional differences, participants in northern and eastern Taiwan reported significantly higher satisfaction than those in central and southern Taiwan, with no significant difference between northern and eastern Taiwan. For quality of care, participants in eastern Taiwan reported significantly higher scores than those in northern and central Taiwan, and participants in northern Taiwan reported significantly higher quality of care than those in southern Taiwan. For enablement, participants in eastern Taiwan reported significantly higher scores than those in central and southern Taiwan, and participants in northern Taiwan reported significantly higher enablement than those in southern Taiwan (all *p*<.001). NEWS 2 scores were found to correlate with lower satisfaction (*p*<.05), quality of care (*p*<.001), and enablement (*p*<.001), indicating an association between higher illness severity and poorer care outcomes. Also, compared with emergency admissions, outpatient admissions were more significantly associated with higher satisfaction (*p*<.001), quality of care (*p*<.01), and enablement (*p*<.001). Participants in the surgical department reported lower satisfaction (*p*<.01), quality of care (*p*<.05), and enablement (*p*<.01) than their peers in internal medicine. In addition, self-perceived health status was strongly associated with all outcome measures (*p*<.001), underscoring the influence of subjective health perception on patient-reported experiences. These results confirm the combined effects of health care setting, care-provider ratios, and patient characteristics on reported outcomes.

### Predictors of Satisfaction, Quality of Care, and Enablement

The predictors of patient satisfaction, quality of care, and enablement identified using multiple linear regression analysis are shown in Tables 3–5. The NP-to-acute-care-beds ratio was identified as a significant positive predictor of patient satisfaction (Table 3; *B* = 18.32, *p* = .031). The participants in southern Taiwan reported lower satisfaction than those in northern Taiwan (*B* = −2.26, *p* = .029); those admitted through the emergency department reported lower satisfaction (*B* = −1.51, *p* = .002) than those admitted through outpatient departments; those in internal medicine reported higher satisfaction than those in the surgical department (*B* = 1.58, *p* = .003); and better self-perceived health status was found to be positively associated with care satisfaction (*B* = 0.78, *p* = .002). Thus, lower NP staffing, living in southern Taiwan, experiencing emergency admission to a surgical department, and having a lower perceived health status all negatively affected satisfaction with care provided by acute care NPs.

**Table 3:** Predictors of Patient Satisfaction (N = 708)

Variable	Patient Satisfaction						
	*B*	β	*SE*	*t*	*p*	95% CI	
						Lower	Upper
NP-to-acute-care-beds ratio	18.32	.10	8.48	2.16	.031^*^	1.69	34.98
Institution							
Regional hospital vs. medical center	−0.16	−.01	1.08	−0.15	.884	−2.27	1.95
Area of residence (ref.: north)							
Central	−1.82	−.09	1.26	−1.44	.150	−4.30	0.66
Southern	−2.26	−.18	1.03	−2.19	.029^*^	−4.29	−0.23
East	−0.25	−.01	0.95	−0.26	.792	−2.11	1.61
NEWS 2	−0.18	−.04	0.17	−1.07	.286	−0.51	0.15
Admission route	−1.51	−.12	0.49	−3.07	.002^**^	−2.47	−0.54
ER vs. outpatient clinic							
Medical division	1.58	.12	0.54	2.95	.003^**^	0.53	2.63
Internal medicine vs. surgical department							
Length of hospital stay	0.04	.05	0.03	1.40	.163	−0.01	0.09
Perceived health status	0.78	.13	0.25	3.09	.002^**^	0.28	1.27

*Note*. NPs = nurse practitioners; ER = emergency room; NEWS 2 = National Early Warning Score 2. Models adjusted for institutional, demographic, and clinical covariates.

* *p* < .05. **p < .01.

**Table 4:** Predictors of Quality of Care (N = 708)

Variable	Quality of Care						
	*B*	β	*SE*	*t*	*p*	95% CI	
						Lower	Upper
NP-to-acute-care-beds ratio	8.50	.10	4.03	2.11	.035^*^	0.60	16.40
Institution							
Regional hospital vs. Medical center	−0.51	−.08	0.51	−1.00	.318	−1.50	0.49
Age	−0.01	−.04	0.01	−0.97	.331	−0.02	0.01
Area of residence (ref.: north)							
Central	−0.27	−.03	0.60	−0.45	.651	−1.45	0.91
Southern	−0.88	−.15	0.48	−1.84	.066	−1.82	0.06
East	0.08	.01	0.45	0.19	.853	−0.80	0.96
NEWS 2	−0.19	−.09	0.08	−2.35	.019^*^	−0.34	−0.03
Admission route	−0.40	−.07	0.23	−1.74	.082	−0.85	0.05
ER vs. outpatient clinic							
Medical division	0.59	.09	0.26	2.29	.023^*^	0.08	1.09
Internal medicine vs. surgical department							
Perceived health status	0.41	.14	0.12	3.43	.001^***^	0.17	0.64

*Note*. NPs = nurse practitioners; ER = emergency room; NEWS 2 = National Early Warning Score 2. Models adjusted for institutional, demographic, and clinical covariates.

* *p* < .05. **p < .01. ***p < .001.

**Table 5:** Predictors of Patient Enablement (N = 708)

Variable	Patient Enablement						
	*B*	β	*SE*	*t*	*p*	95% CI	
						Lower	Upper
NP-to-acute-care-beds ratio	11.49	.11	4.90	2.34	.019^*^	1.87	21.11
Institution							
Regional hospital vs. medical center	−1.19	−.16	0.63	−1.95	.052	−2.39	0.01
Sex							
Female vs. male	0.52	.07	0.27	1.90	.058	−0.02	1.06
Age	−0.01	−.05	0.01	−1.09	.277	−0.03	0.01
Educational level (ref.: elementary school or below)							
Junior high school	−0.37	−.04	0.45	−0.82	.410	−1.25	0.51
High school	0.12	.02	0.42	0.28	.776	−0.71	0.94
College or above	0.03	.00	0.45	0.06	.955	−0.86	0.91
Area of residence (ref.: north)							
Central	0.30	.03	0.72	0.42	.674	−1.12	1.73
Southern	−0.42	−.06	0.58	−0.72	.471	−1.56	0.72
East	−0.40	−.04	0.55	−0.72	.473	−1.48	0.69
NEWS 2	−0.22	−.09	0.10	−2.28	.023^*^	−0.41	−0.03
Admission route	−0.87	−.12	0.28	−3.11	.002^**^	−1.40	−0.32
ER vs. outpatient clinic							
Medical division	0.67	.09	0.31	2.17	.030^*^	0.06	1.28
Internal medicine vs. surgical department							
Perceived health status	0.53	.15	0.14	3.68	.001^***^	0.25	0.81

*Note*. NPs = nurse practitioners; ER = emergency room; NEWS 2 = National Early Warning Score 2. Models adjusted for institutional, demographic, and clinical covariates.

* *p* < .05. **p < .01. ***p < .001.

The regression results identified the NP-to-acute-care-beds ratio as a positive predictor of quality of care (Table 4; *B* = 8.50, *p* = .035), with higher NEWS 2 scores (representing greater patient acuity) associated with lower quality of care (*B* = −0.19, *p* = .019). The participants in internal medicine reported a higher quality of care than those in the surgical department (*B* = 0.59, *p* = .023). Self-perceived health status demonstrated a strongly positive association with quality of care (*B* = 0.41, *p* = .001). These results highlight the key influences of NP staffing, patient acuity, medical division, and perceived health status on patient-perceived quality of care.

The NP-to-acute-care-beds ratio was identified as a positive predictor of enablement (Table 5; *B* = 11.49, *p* = .019), with higher NEWS 2 scores associated with lower enablement (*B* = −0.22, *p* = .023). Compared with outpatient admission, admission through an emergency department was associated with lower enablement (*B* = −0.87, *p* = .002). Also, those with higher perceived health status reported relatively higher enablement (*B* = 0.53, *p* = .001). In addition, participants in internal medicine reported higher enablement than those in the surgical department (*B* = 0.67, *p* = .030). These findings identify the NP-to-acute-care-beds ratio, admission route, medical division, and perceived health status as key predictors of patient enablement.

PESS scale scores demonstrated strong internal consistency, with mean scores of 43.35 (*SD* = 6.30) for patient satisfaction, 16.88 (*SD* = 3.00) for quality of care, and 21.35 (*SD* = 3.61) for patient enablement. Each dimension correlated highly with the total score (*r* = .79–.85, *p* < .01), supporting the conceptual coherence and construct validity of this instrument.

## Discussion

The sample used in this study comprised 708 patients hospitalized in the acute care wards of 10 major medical institutions, including three large medical centers and seven regional hospitals, across Taiwan. The PESS was administered to evaluate three key outcomes of NP care, including patient-perceived satisfaction, quality of care, and enablement. The participants self-reported the care received from NPs as being highly satisfactory, high quality, and enabling. NP-to-acute-care-beds ratio, medical division, emergency admission, living in southern Taiwan, and perceived health status were all shown to significantly influence satisfaction with NP care. The analysis results showed the significant predictors of quality of care to be the NP-to-acute-care-beds ratio, acute disease severity, medical division, and self-perceived health status, and those of enablement to be the NP-to-acute-care-beds ratio, acute disease severity, emergency admission, medical division, and perceived health status.

Both high patient satisfaction with the care provided by NPs and the key factors influencing patient-reported satisfaction, quality of care, and enablement were revealed in this research, which was the first multi-hospital investigation in Taiwan to measure NP care outcomes with a reliable and validated instrument. Within Taiwan’s health care system, NPs have provided direct care to hospitalized patients for more than two decades. This study was the first conducted in Taiwan to report a high level of satisfaction among acute care patients with the care provided by NPs. Moreover, the high levels of satisfaction, quality of care, and enablement observed among the patients in this study are comparable to outcomes reported in studies conducted in Australia and the United States (Desborough et al., 2016; McConkey et al., 2023; O’Reilly et al., 2021). These findings further support the contribution of NPs toward the achievement of satisfactory clinical outcomes among hospitalized patients.

Maintaining a higher NP-to-acute-care-beds ratio was identified as a vital predictor of patient-perceived satisfaction, quality of care, and enablement. This finding aligns with a growing body of evidence supporting the expansion of NP roles and staffing in acute care settings (Aiken et al., 2021; John et al., 2024). Adequate NP staffing improves clinical outcomes and supports patient-centered care, which are critical indicators of health care quality. In addition, higher NP staffing in hospitals benefits patients, staff nurse satisfaction, and quality of care, while also decreasing workload pressure and burnout among NPs (John et al., 2024). Thus, high NP staffing ratios add value for hospitals, patients, and nurses.

In Taiwan, regional disparities in health care access and quality influence patient satisfaction and care outcomes considerably. In this study, the participants in southern Taiwan generally reported poorer care outcomes than their peers in other areas, likely due to the fewer health care resources there than elsewhere (Yaya et al., 2017). Patient-centered care, which involves effective communication, information sharing, and emotional support, is particularly critical to patient satisfaction, especially in chronic care settings (Chen et al., 2023). Type of institution (i.e., medical center vs. regional hospital) was found to have no significant influence on patient-perceived satisfaction, quality of care, or enablement. These results may be reflective of the fact that NPs provide patient-centered care and thus not only explain detailed information related to patients’ disease management but also educate patients on effective strategies for adapting to their illness. However, the relatively small number of institutions (three medical centers and seven regional hospitals) surveyed in this study may not fully capture the full spectrum of health care institutions in Taiwan. Although Taiwan’s National Health Insurance system provides comprehensive coverage, equity in health care quality remains a pressing challenge, particularly in the country’s southern region. Targeted policy interventions are needed to address resource disparities and improve health care equity (Sehgal et al., 2024; Yaya et al., 2017).

Type of admission was shown to significantly affect satisfaction and enablement in this study, with those admitted through the emergency department reporting lower average satisfaction than those admitted electively. Emergency admissions are often correlated with overcrowding (Pines et al., 2008), prolonged waiting times (Lewis Hunter et al., 2016), and hallway care (Pines et al., 2008), which are known to contribute to negative care experiences. Also, the unexpected nature of emergency admissions increases patient anxiety and reduces their sense of preparedness (Fenton et al., 2014). Moreover, the limited time available for patient education during rushed emergency care may hinder the ability of patients to understand and manage health concerns effectively (i.e., enablement; Ämmälä & Taimela, 2024). Conversely, elective admissions are associated with higher satisfaction (Fenton et al., 2014). Patients can prepare mentally and logistically for planned hospitalizations, fostering a more positive experience (Fenton et al., 2014). Continuity with familiar health care providers promotes communication and trust, while the ability to choose the timing of admission gives patients a greater sense of control over their health care journey (Fenton et al., 2014). Opportunities for education and preparation before an elective admission improve patient enablement and outcomes (Ämmälä & Taimela, 2024; Fenton et al., 2014).

Patients receiving care from NPs in internal medicine wards reported higher satisfaction and quality of care than those in the surgical department. The typically longer hospitalization duration and more intensive provision of NP care among patients with chronic illness may explain this finding. Perceived health status was also identified as a key predictor of satisfaction, quality of care, and enablement. In prior studies, patients with better self-rated health have consistently reported higher health care satisfaction. For example, patients with “excellent” or “good” health scores reported greater access to care, provider quality, and overall quality of care (Beyene et al., 2021; Suhonen et al., 2018). In another study, patients whose health was classified as poor were 1.5 times less likely than other patients to report high care satisfaction (Kingston et al., 2023). Perceived health status may also affect perceived quality of care, with higher ratings linked to better evaluations of provider performance and care standards (Beyene et al., 2021; Suhonen et al., 2018).

Patient enablement, that is, the ability of patients to self-manage their health, is also affected by perceived health status. Chronic illness and poor self-perceived health status correlate with lower enablement, and higher enablement has been shown to mitigate the negative effects of chronic pain on health-related quality of life (Ng et al., 2023). Enablement is also linked to self-reported health improvements. These findings underscore the critical role of perceived health status in shaping health care experiences and outcomes. Patients with more positive perceptions of their health tend to report greater satisfaction, quality of care, and enablement, indicating subjective health perceptions must be addressed during health care evaluations (Ämmälä & Taimela, 2024; Beyene et al., 2021; Kingston et al., 2023).

### Limitations and Future Recommendations

This study was affected by several limitations. First, the cross-sectional study design limits causal inference, and the sample of 10 health care institutions may not adequately represent the diversity of health care settings in Taiwan or other Asian countries. In particular, the underrepresentation of institutions located in rural areas highlights a need for more comprehensive regional studies. Second, self-selection bias, inherent to survey studies, may have encouraged greater participation from patients who were satisfied with their care. Future studies with longitudinal designs and broader sampling scopes should be conducted to provide deeper insights. Third, as the modified PESS is a reasonably new measure, the reliability and validity of this tool should be further investigated and confirmed.

Hospitals should increase NP staffing in underserved areas, address urban–rural disparities in health care access, and further tailor communication strategies and resources to strengthen patient-centered care. These measures can increase satisfaction, quality of care, and enablement, contributing to more equitable and effective health care delivery. Because the large sample size (*n* = 708) in this study significantly exceeded the minimum threshold (*n* = 152), the results may have been influenced by overpowering, which may limit the clinical interpretability of the findings. However, the validity of the findings remains uncompromised, as larger sample sizes enhance the robustness of the results and diminish the likelihood of Type II errors (Lakens, 2024).

### Conclusions/Implications for Practice

All of the patients receiving acute care from NPs in this study reported high levels of satisfaction, quality of care, and enablement. Variables including the NP-to-acute-care-beds ratio, area of residence, medical division, admission route, and perceived health status were identified as significantly influencing patient satisfaction with NP care (*p* < .05). Also, the NP-to-acute-care-beds ratio, acute disease severity, medical division, and self-perceived health status were all shown to significantly predict quality of care (*p* < .05), while the NP-to-acute-care-beds ratio, acute disease severity, admission route, medical division, and perceived health status were shown to significantly predict patient enablement (*p* < .05). These findings support previous research in highlighting the contribution of NPs to direct health care for hospitalized patients. The findings of this study offer vital information for acute care NPs and hospital administrators regarding strategies aimed at improving satisfaction, quality of care, and enablement among patients receiving care from NPs.
